# Evidence for a centrosome-attracting body like structure in germ-soma segregation during early development, in the urochordate Oikopleura dioica

**DOI:** 10.1186/s12861-018-0165-5

**Published:** 2018-02-27

**Authors:** Lisbeth Charlotte Olsen, Ioannis Kourtesis, Henriette Busengdal, Marit Flo Jensen, Harald Hausen, Daniel Chourrout

**Affiliations:** 1 Sars International Centre for Marine Molecular Biology, University of Bergen, Bergen, Norway; 2 Department of Molecular Biology, University of Bergen, Thormöhlensgt 55, 5008 Bergen, Norway; 3 Department of Biological Sciences, University of Bergen, Thormöhlensgt 55, 5008 Bergen, Norway

**Keywords:** Centrosome attracting body, *Pumilio*, *Vasa*, Postplasmic/PEM transcripts, Early development, Cell fate, Tunicates

## Abstract

**Background:**

Germ cell formation has been investigated in sessile forms of tunicates. This process involves the release of a subset of maternal transcripts from the centrosome-attracting body (CAB) in the progenitor cells of the germ line. When germ-soma segregation is completed, CAB structures are missing from the newly formed primordial germ cells (PGCs). In free-swimming tunicates, knowledge about germ cell formation is lacking. In this investigation, comparative gene expression and electron microscopy studies were used to address germ cell formation in *Oikopleura dioica* (*O. dioica*).

**Results:**

We found that the RNA localization pattern of *pumilio* (*pum1*) is similar to the pattern described for a subset of maternal transcripts marking the posterior end of ascidian embryos. Transcripts marking the posterior end are called postplasmic or posterior-end mark (PEM) transcripts. We found no localization of *vasa* (*vas*) transcripts to any sub-region within the germ-line precursor cells. Expression of *vas4* was detected in the newly formed PGCs. Electron microscopy studies confirmed the presence of structures with similar morphology to CAB. In the same cytoplasmic compartment, we also identified *pum1* transcripts and an epitope recognized by an antibody to histone H3 phosphorylated on serine 28.

**Conclusions:**

Our findings support that a CAB-like structure participates in the segregation of maternal *pum1* transcripts during germ-soma separation in *O. dioica.*

**Electronic supplementary material:**

The online version of this article (10.1186/s12861-018-0165-5) contains supplementary material, which is available to authorized users.

## Background

### Germ line development in solitary ascidians

One of the challenges in developmental biology is to understand how cells adopt specific characteristics during embryogenesis. One mechanism is asymmetric cleavages and unequal segregation of localized cytoplasmic factors. This is seen in ascidian embryos, which develop in a typical mosaic manner, where maternally supplied factors control cell fate specification reviewed by [1]. For example, in the ascidian *Ciona intestinalis,* several maternal transcripts are transiently localized to the vegetal pole of fertilized eggs [2]. As development proceeds, maternal transcripts move to the future posterior pole. These transcripts together with cortical endoplasmic reticulum (cER) and mitochondria form the posterior vegetal cytoplasm/cortex (PVC), also called postplasm [3]. During subsequent steps of embryogenesis, the PVC segregates along with the posterior blastomeres. During this process, the cER domain with its associated localized transcripts (classified as postplasmic or posterior end mark (PEM) transcripts) and proteins condense into a macroscopic structure. This structure is called the centrosome-attracting body (CAB), which is first detectable in the B4.1 blastomeres of 8-cell stage embryos [2]. The CAB structure also contains germ plasm components [4] and participates in the unequal cleavages of the posterior blastomeres located in the vegetal hemisphere (B4.1, B5.2, B6.3, B7.6) from the 8-cell stage to the gastrulation stage. When the B7.6 blastomeres divide, they produce two distinct populations of daughter cells, two primordial germ cells (B8.12) and two endodermal strand cells (B8.11) [4]. During this cell division, postplasmic/PEM transcripts have distinct cell destinations [5]). One subset of postplasmic/PEM transcripts, still attached to the CAB, segregate into the endodermal strand cells (B8.11). One of the important gene in this group is *posterior-end mark* (*pem-1*). PEM-1 has a role in repressing gene expression in the germ line precursor cells during germ-soma segregation [6, 7]. Another set of postplasmic/PEM transcripts includes *vasa* (*vas*) transcripts [4]. *vas* is a well-known germ cell marker. In ascidian embryos, *vas* transcripts are released from the CAB located in the germ line precursor B7.6 blastomeres. Both the PGCs (B8.12 cells) and the endodermal strand cells (B8.11 cells) inherit these transcripts.

### Germ line development in free-swimming tunicates

Comparatively little is known about how PGCs are formed in larvaceans. The first descriptions of early embryogenesis of the larvacean, *Oikopleura dioica (O.dioica),* date back to the early twentieth century [8]. Delsman described the early cleavage pattern of fixed samples of *O. dioica* embryos, from the first to the sixth cleavage. A century later, Stach and co-workers presented the first detailed cell lineage map, which was based on direct observations of living *O. dioica* embryos combined with 4D microscopy [9]. In addition, Fujii and co-workers reported the early cleavage pattern of live *O. dioica* embryos up to the gastrulation stage [10]. The cleavage pattern described in the two recent studies is mostly consistent with the descriptive findings of Delsman. One exception is the ‘*two plasma hump cells’*, which Delsman indicated to be of animal origin and located at the posterior pole of early embryos. These cells were actually shown to be of vegetal origin in the work of both Stach and Fujii [9, 10]. According to both descriptions of the cell lineage map, the posterior-most localized blastomeres, B6.4, are the founder cells of the germ line (see Fig. 1 in [9]), or potentially the PGCs [10]. The position of these cells corresponds to the position of the ‘two *plasma humps’,* described by Delsman (1910). The reasoning behind B6.4 cells being PGCs was that the cleavage pattern of the posterior-vegetal B-line in *O. dioica*, from the 8-cell stage up to the 32- cell stage, appears similar to the *unequal cleavages* taking place at the posterior pole of ascidian embryos during cleavage stages [10]. As development proceeds, the two presumptive PGCs, the B6.4 cells, ingress and become positioned in the posterior trunk of the embryo [10].

**Fig. 1:**
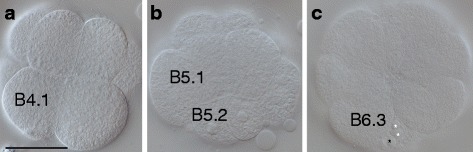
Description of early embryogenesis in *O. dioica*. The developmental stages included are: vegetal views of an 8 cell-stage embryo (**a**), a 16-cell stage embryo (**b**), and an embryo which has completed stage one of gastrulation (**c**). The gastrulation stage shown here corresponds to the transition from 32-cell to 62-cell stage. This stage is easily recognizable due to the star-like organization of the blastomeres on the vegetal surface. The nomenclature is according to the nomenclature of Conklin (see [9]). B4.1 and B5.2 are germ line precursor cells. The small B5.2 cell and the large B5.1 cells are daughter cells of the germ line precursor cell B4.1. The white asterisks mark each of the two detected refractive structures within one of the B6.4 cells. The black asterisk marks one observed refractive structure in the other B6.4 cell. The small sized germ line cell B6.4 and the larger somatic B6.3 blastomeres are sibling cells and daughter cells of the germ line precursor cell B5.2 [9, 10]. Scale bar: 50 μm

To explore the steps in germ cell specification in *O. dioica*, we used a candidate gene approach to search for maternal factors that may be involved in the early patterning of the embryo. This led to the identification of the homologs of *pumilio* (*pum*) and *vas* in *O. dioica.* The PUM protein is a member of the PUF family [11] of conserved RNA-binding proteins, which are factors involved in regulating many developmental processes by controlling mRNA stability or translation. Among the processes associated with *pum* are: anterior-posterior patterning of the embryo, germ line development, and regulation of asymmetric divisions of germ line stem cells [12, 13]. Among several other species, *vas* homologs serve as markers for the lineage giving rise to the PGCs. For example, in *C. intestinalis* both *vas* transcripts and Vas protein have been detected within the CAB region [13].

This study is the first one to reveal the presence of a postplasm in *O. dioica*. The two postplasmic components identified are maternal *pum1* transcripts and an epitope recognized by an antibody to histone H3 phosphorylated on serine 28. The cell type segregation pattern for *pum1* is similar to that described for *pem-1* in ascidians. We have also characterized the expression pattern of four identified *vas* genes. Our in situ analyses did not reveal postplasmic localization in the presumptive germ line blastomeres for any of the maternal *vas* transcripts. However, zygotic expression of the *vas4* gene was detected in newly formed PGCs. We also identified CAB-like structures in the presumptive germ line blastomeres. On the basis of these new data, we provide a new model for the segregation of the germ line in *O. dioica.*

## Results

### Early embryogenesis and distinctive features

Images of the developmental stages for early *O. dioica* embryogenesis that were important for this investigation are presented in Fig. 1. The cleavage pattern of the presumptive germ line blastomeres (B4.1 and B5.2), which are located at the posterior pole in the vegetal hemisphere, has been previously described [10]. These blastomeres undergo unequal cleavages so that the smallest daughter cells are always situated at the posterior pole. During the gastrulation stage, the presence of two large, refractive structures in the germ line blastomeres B6.4 were detected by light microscopy (Fig. 1c). This finding is consistent with Delsman’s description of these cells [8].

### Maternal *pum* transcripts mark the posterior end of *O. dioica* embryos

**Fig. 2:**
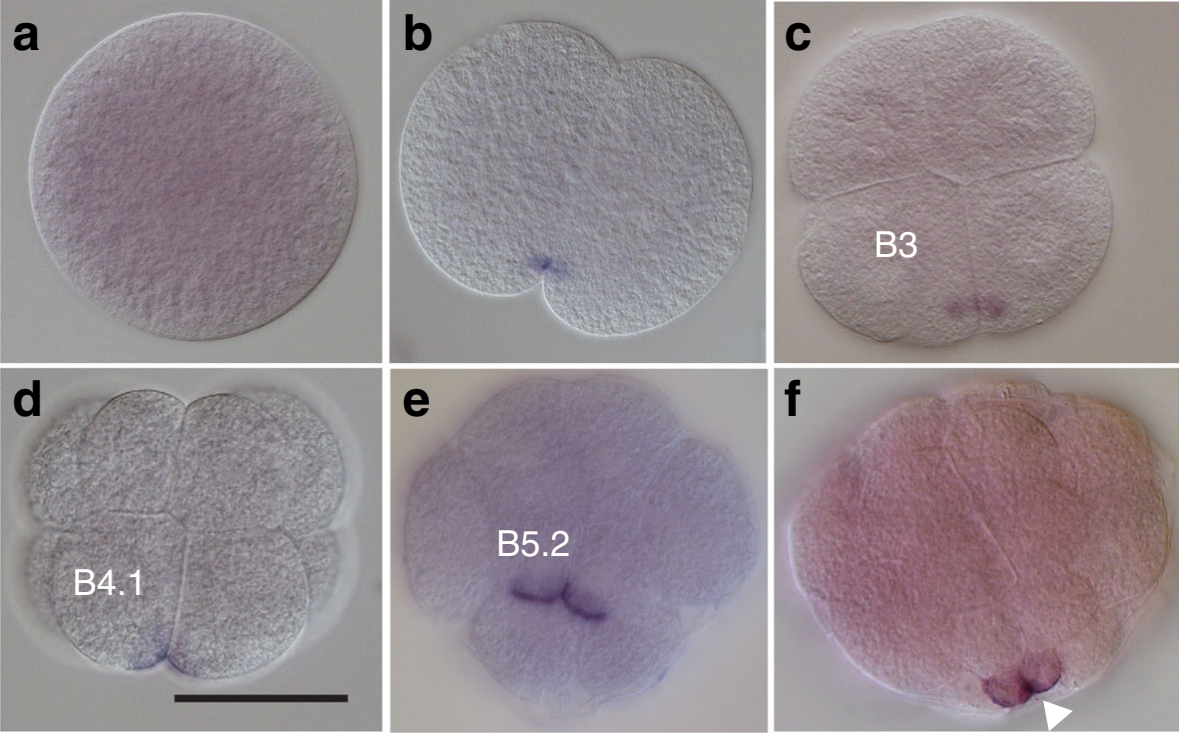
*pum1* transcripts mark the posterior end of *O. dioica* embryos. In situ hybridization analysis was performed with an antisense *pum1* probe. The developmental stages included are: fertilized egg (**a**), 2-cell stage (**b**), 4-cell stage (**c**), 8-cell stage (**d**), 16-cell stage (**e**), and 32-cell stage embryos (**f**). *pum1* transcripts are tightly localized to one side of the cell and found in close proximity to the plasma membrane. These transcripts are detected in the cell lineage which gives rise to the germ line (B4.1, B5.2) and the germ line blastomeres (B6.4) [9]. The white triangle points to the B6.4 cells, the PGCs, according to the fate map. Scale bar: 50 μm

*O. dioica* has two *pum* genes and they are organized in two separate operons. Both genes are maternally expressed, consistent with the tiling-array expression data in the OikoBase [14]. While no obvious signal was obtained with the *pum2* RNA probe during early embryogenesis, *pum1* transcripts became localized during embryogenesis (Fig. 2). Specifically, in unfertilized eggs, *pum1* transcripts were homogenously distributed (Fig. 2a). At the 2-cell stage, *pum1* transcripts were localized/enriched to one end along the anterior-posterior axis (see Fig. 2b). In the four-cell stage embryos, *pum1* transcripts were tightly restricted to the cortex of the posterior region of the B3-blastomeres (Fig. 2c). At the 8- and 16-cell stages, *pum1* transcripts were organized in a crescent-like pattern in the most posterior region of the B4.1 and B5.2 blastomeres, respectively (Fig. 2d-e). *pum1* transcripts were localized to the sub-cortex of the B6.4 cells of a 32-cell stage embryo (Fig. 2f). Thus, *pum1* transcripts mark the posterior-most region of the embryos and are part of the cell-lineage giving rise to PGCs. The observed in situ pattern of *pum1* is very similar to the localization patterns described for postplasmic/PEM transcripts in ascidians [5, 15, 16]. Because germ line precursor cells are transcriptionally repressed [17], we suggest that the identified *pum1* transcripts are of maternal origin.

### Electron microscopy studies reveal CAB-like structures in the precursor germ cells of *O. dioica*

**Fig. 3:**
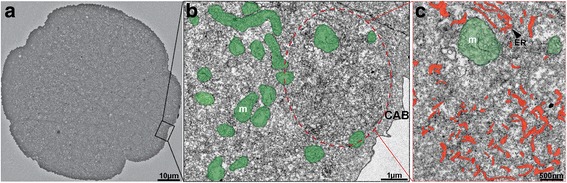
Ultrastructural identification of a CAB-like region in *O. dioica.* Representation of a 16-cell stage embryo at low magnification highlighting the *pum1* mRNA rich region of the germ line precursor blastomere B5.2, the area enclosed by the rectangle (**a**). The circle encloses the CAB-like region, identified by a high density of rough ER and accumulation of mitochondria in the surrounding region (**b**). Higher magnification of the CAB-like region shows relatively high accumulation of rough ER (**c**). Mitochondria are labeled in green. ER is marked in red

The CAB region has been previously morphologically characterized by electron microscopy studies in ascidians. This region was shown to be highly enriched in the sub-cortical endoplasmic reticulum (cER) compared to the surrounded region enriched in mitochondria [2, 18]. To find support for a CAB-like structure in the precursor germ line cells in *O. dioica*, transmission electron microscopy was performed across the region corresponding to the *pum1* mRNA domain of a 16-cell stage embryo. Since the CAB structure is expected to have a depth of 4–8 μm [2], we decided to go through a series of ultrathin (70 nm) sections through the whole embryo in order to identify the region of interest (Fig. 3a). Subsequent examination at higher resolution revealed high density of rough ER in an area spanning 5–8 μm and accumulation of mitochondria in the surrounding region (Fig. 3b and c). These observations strongly indicate that *O. dioica,* as in ascidians, possesses CAB-like structures in the germ line precursor cells.

### The fate of maternal *pum1* transcripts

**Fig. 4:**
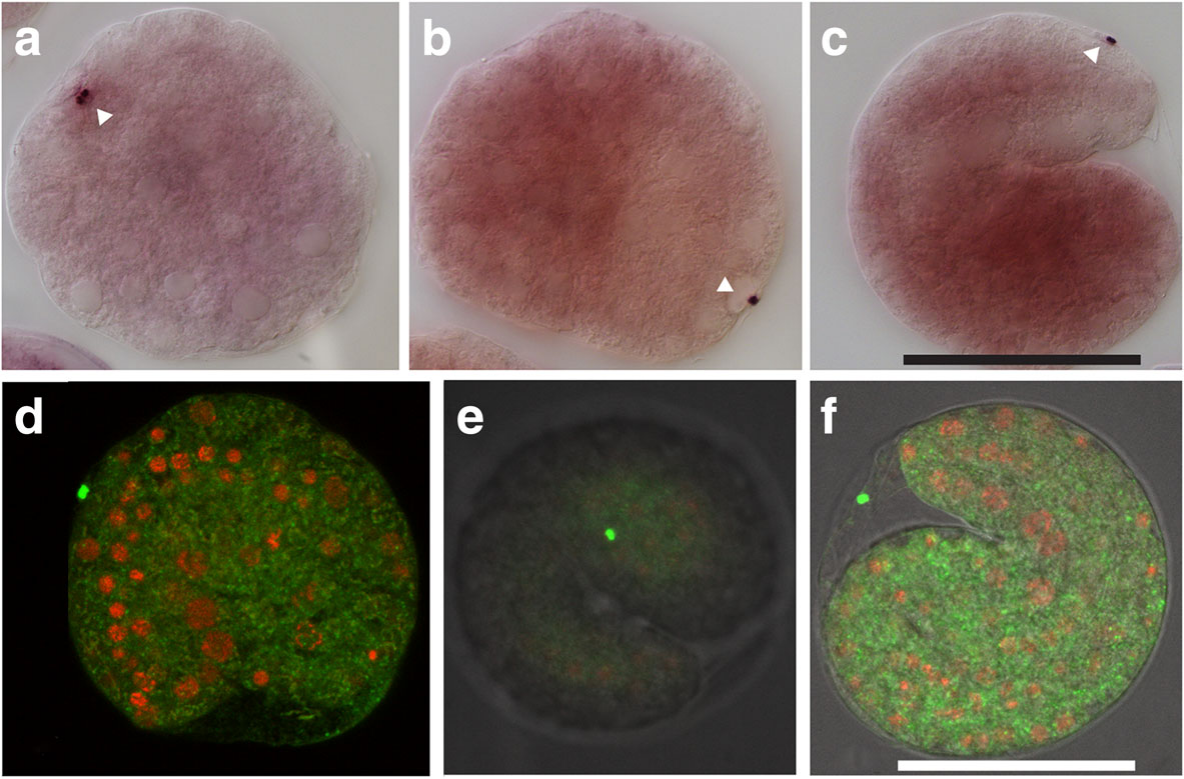
*pum1* is not a PGC marker in *O. dioica.* In situ hybridization with a DIG-labeled RNA probe showed that *pum1* transcripts remain localized to the surface of the developing embryos from gastrulation stage (panels **a**-**c**). In situ labeling with a fluorescent-labeled RNA probe revealed detection of *pum1* signal outside of the embryos prior to hatching (panels **d**-**f**). Nuclei (red signal) were visualized by DAPI staining. The developmental stages included are: late gastrulation stage (**a**), early tailbud (2.5 h post-fertilization) (**b**), and close to hatching (**c**) - (**f**). The white triangle points to the *pum1* signal. Scale bar: 50 μm

Stach and co-workers have reported that after formation of the B6.4 cells, they arrest cleavage, ingress and become situated in the posterior trunk [9]. Our in situ analysis shows that *pum1* transcripts are detected in cells located at the surface of the embryo, rather than inside ingressed cells of the embryo where we expect to find PGCs at these stages of development (Fig. 4). Therefore, *pum1* is not a PGC marker in *O. dioica*.

While following the maternal *pum1* transcripts through further stages of development, we were surprised to detect them outside the embryo prior to hatching (Fig. 4d-f, Additional file 1). We consider two plausible interpretations for this observation: either maternal *pum1* transcripts still attached to a CAB-like structure have been released from the cells, or the *pum1* transcripts are actively being secreted from the cells.

### Progression of the B6.4 cells through mitosis during gastrulation

**Fig. 5:**
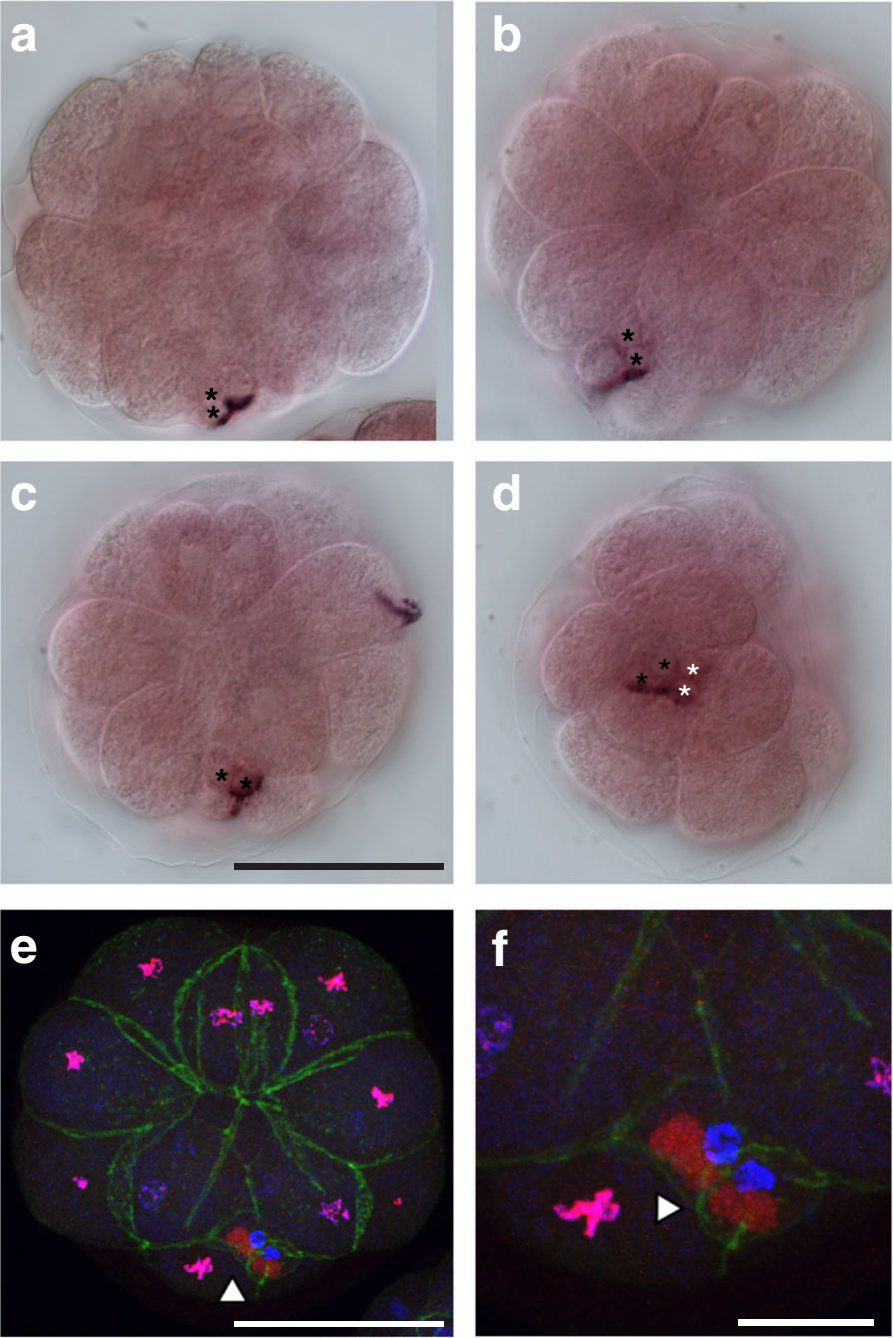
Detection of mitotic B6.4 cells during gastrulation. Localization of *pum1* transcripts in embryos at gastrulation stage (panels **a** to **d**) and confocal images of an embryo stained for phosphorylated histone H3, a mitosis-specific marker (panels **e** to **f**). In panels **a** to **d** are shown representative images of different embryos at gastrulation stage hybridized with a DIG-labeled *pum1* RNA probe. Vegetal (**a** to **c**) or posterior (**d**) views are shown. *pum1* expression is detected in each of the B6.4 cells. Two nuclei (marked with black asterisks) are observed in one of the two B6.4 cells of the embryos shown in panels **a** to **c** (*note:* the nuclei of the other B6.4 cell are out of the focal plane in these three panels). In panel **d**, however, two nuclei are seen in both B6.4 cells (marked with white and black asterisks, respectively). In panel **e** is shown a vegetal view of an embryo labeled for phosphorylated histone H3 (phosphorylated serine 28, H3S28p; red). In overlay is also shown labeling for microfilaments (green) and DNA (blue). In all cells except the B6.4 cells, chromosomes appear pink due to the overlay of DNA and H3S28p staining. In panel **f** is shown a close-up view of the B6.4 cells with heavily condensed chromosomes (blue). In these B6.4 cells, the anti-H3S28p antibody stained a large non-chromosomal subcellular domain (red; marked by white arrow heads). Scale bar is 50 µm in panel E and 15 µm in panel **f**.

Our in situ analysis revealed the presence of *pum1* transcripts in B6.4 cells containing a pair of nuclei at late gastrulation stage (Fig. 5a-d, Additional file 2). We have also noticed the presence of two relatively large structures in the B6.4 cells (Fig. 1c). These observations are in line with the description Delsman gave of the B6.4 cells during gastrulation [8]. Our data support the idea that these cells are progressing through cytokinesis during late gastrulation.

Serine 28 phosphorylation of histone H3 (H3S28) is associated with chromosome condensation during mitosis [19]. Because H3S28 phosphorylation serves as a mitosis marker, we examined whether an H3S28 antibody would recognize phosphorylated chromosomes in the B6.4 cells (Fig. 5e and f). Although we did detect mitotic cells in the embryo at the gastrulation stage, no signal was observed on the chromosomes in the B6.4 cells (Fig. 5e and f). However, the DAPI staining revealed the presence of heavily condensed chromosomes in the B6.4 cells (Fig. 5e-f). This finding indicates that these cells, during early gastrulation, have already passed the metaphase of mitosis, a stage where de-phosphorylation of histone H3 is known to be completed [19]. Interestingly, in each of the B6.4 cells, the H3S28 antibody stains a large, non-chromosomal subcellular domain, located just beneath the cell membrane (Fig. 5e and f). We believe this structure is the CAB-like structure. With this antibody, a similar staining was also observed in germ line precursor cells at earlier stages of development (see Additional file 3).

### Zygotic transcription of vas genes in PGCs

**Fig. 6:**
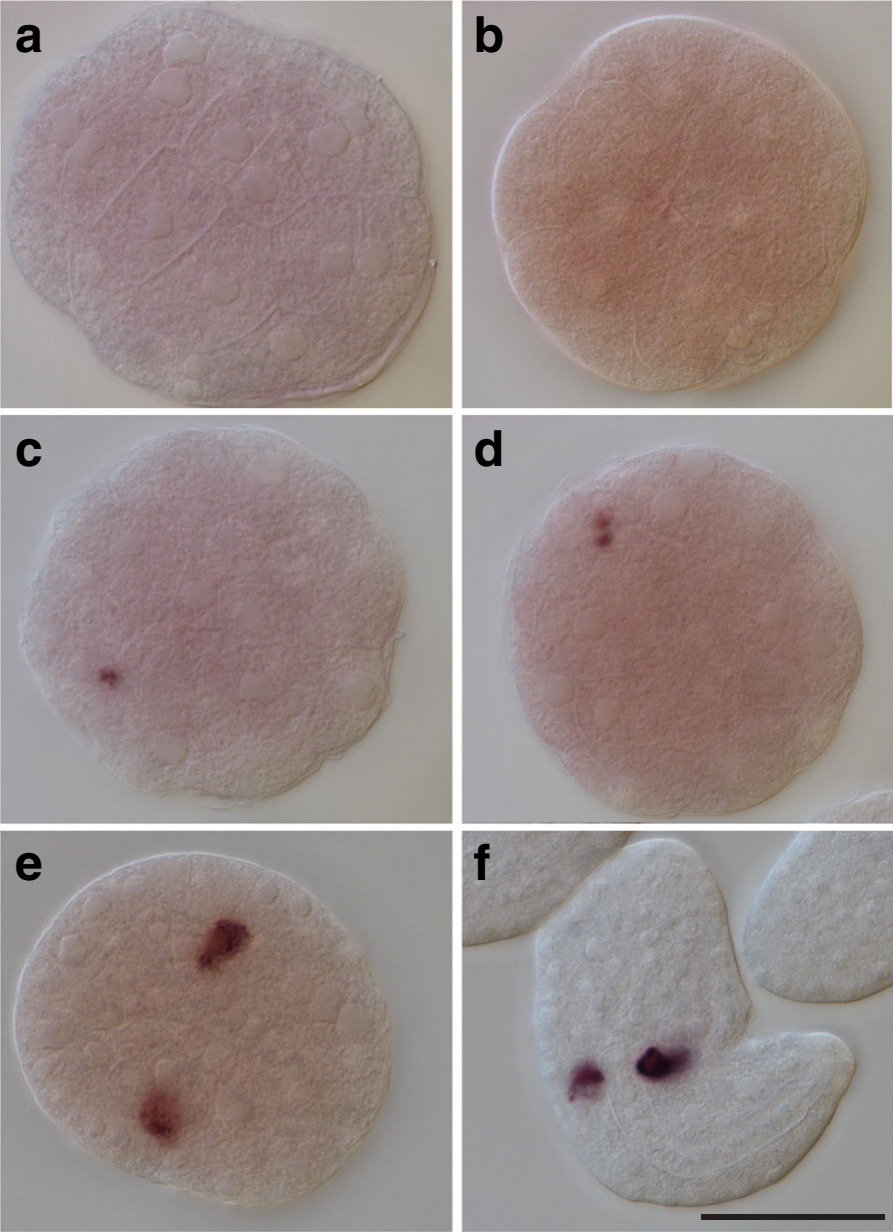
Detection of zygotic *vas4* transcripts in PGCs. In situ hybridization with a DIG-labeled RNA probe detected no signals in embryos at early gastrulation stage (**a**, **b**, vegetal views). Shortly after gastrulation was completed *vas4* transcripts were detected in one or two cells (**c**, **d**). At tailbud stage (**e**) and newly hatched larva (**f**), signals were observed in the posterior trunk. The position of *vas4*-positive cells corresponds to the location of PGCs. Scale bar: 50 μm

The DEAD-box RNA helicase Vas is a conserved germ cell marker in many organisms [20]. We have identified four *vas* genes in *O. dioica* and explored their expression patterns. In situ analyses revealed very weak uniform staining during early embryogenesis for *vas4* (Fig. 6a and b)*,* while no obvious signal was observed for *vas1*, *vas2* and *vas3* at these stages. Hence, *vas* transcripts cannot be classified as postplasmic/PEM transcripts in *O. dioica.*

Shortly after gastrulation was completed, *vas4* transcripts were first detected in one cell, then in two cells (Fig. 6c and d). The position of these cells corresponds with the position in the embryo where we would expect to find the PGCs [9]. In tailbud embryos and hatched embryos, the *vas4*-positive cells are located in the posterior trunk (Fig. 6e and f). Our findings indicate that zygotic transcription of *vas4 is* initiated in PGCs shortly after these cells have been formed.

## Discussion

### Postplasmic components and CAB-like structures in *O. dioica*

Embryonic germ line determination studies are sparse for the free-swimming tunicate *O. dioica* as opposed to the solitary ascidians. In this investigation, our goal was to explore germ-soma segregation in *O. dioica.* A shared feature of larvacean and ascidian early embryonic development is the unequal cleavages of the presumptive germ line blastomeres, the B cells, in the posterior-vegetal hemisphere of the embryo [10]. In ascidians, the CAB structure has a role in regulating the unequal cleavages of these cells in addition to participating in the segregation of maternal postplasmic components (for a review see [21]). We found evidence for the presence of postplasmic transcripts and morphological structures similar to CAB in the presumptive germ line blastomeres of the larvacean species, *O. dioica*. In these cells, the CAB-like structure is stained by the antibody to phosphorylated H3S28. Whether this epitope is phosphorylated H3S28 or another protein remains unclear. We suggest that the CAB-like structure we have identified in the presumptive germ line blastomeres in *O. dioica* has similar roles as has been described for the CAB structure in ascidians.

### *pum1* may control the unequal cleavages of the presumptive germ line blastomeres

One of the identified postplasmic transcripts in ascidians is *pem-1* appears to be ascidian-specific [5, 15]. Morpholino knockdown experiments inhibiting translation of *pem-1* mRNA abolished the unequal cleavages of the germ line blastomeres [22]. We also know that *pum* is not among the identified postplasmic transcripts in ascidians [5]). Because *pum* controls the asymmetric cleavages of germ line stem cells in the female gonad of *Drosophila* [13], we suggest that *pum1* also controls the unequal cleavages of the germ line blastomeres (B4.1, B5.2, and B6.4) in *O. dioica*.

### Germ cell formation and activation of zygotic transcription

It is generally thought that global transcription is repressed in the germ line precursor cells until PGCs are formed [17]. The current view is that newly formed PGCs switch from primarily posttranscriptional to transcriptional gene regulation [23]. This event is concomitant with the elimination of maternal transcripts from PGCs during the maternal-to-zygotic transition (MZT) [23]. In ascidians, PEM-1 is thought to act as a repressor of zygotic transcription in germ line precursor cells [6, 7]. The outcome of germ-soma segregation is that newly formed PGCs, which are lacking the CAB structures initiate zygotic transcription. In contrast, CAB remnants with associated maternal *pem-1* transcripts as well as other postplasmic transcripts end up in the endodermal strand cells.

We suggest that a similar process also occurs during germ cell formation in *O. dioica*. Our reasoning is based on the following findings: (i) we have detected the presence of two nuclei in each of the *pum1*-positive B6.4 cells during late gastrulation, which is in accordance with Delsman’s descriptions [8], (ii) we have found that maternal *pum1* transcripts are present in the two somatic cells (the B7.s) but absent from the PGCs (the B7.g) when gastrulation is completed, and (iii) we have detected the presence of zygotic transcripts of *vas 4* in newly formed PGCs. We suggest that germ-soma segregation has come to an end when the B6.4 cells have progressed through mitosis.

**Fig. 7:**
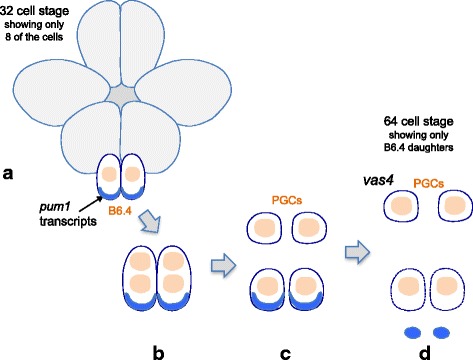
Model for germ line determination in *O. dioica*. This model is based on our in situ data for *pum1* and *vas 4*. The model emphasizes how germ line determination may take place during gastrulation. **a**) Schematic representation of an embryo at the early gastrulation stage. The position of the small B6.4 cells containing maternal *pum1* transcripts are indicated. **b**) A close up of B6.4 cells that have embarked on mitosis during gastrulation. This stage corresponds to the transition from a 32-cell to a 64-cell stage embryo. Each of the cells now contain a pair of nuclei and maternal *pum1* transcripts. **c**) Schematic representation of the outcome of the division of the B6.4 cells. Two PGCs lacking maternal transcripts and two somatic cells containing maternal *pum1* transcripts are depicted. At this stage of development germ-soma segregation is completed. **d**) The two PGCs depicted initiate expression of the germ cell marker *vas4* when they have ingressed during gastrulation. The two somatic cells depicted have lost the maternal *pum1* transcripts and they do not ingress during gastrulation. The circumference of the two somatic cells is shown with dashed lines indicating that these cells may undergo apoptosis

Our current working model emphasizes how germ line formation may take place during gastrulation (Fig. 7). In this model, the germ line precursor B6.4 *pum1* positive cells undergo asymmetric cell cleavages leading to the formation of two PGCs and two somatic cells. The somatic cells (B7.s) inherit CAB-like structures with maternal *pum1* transcripts associated to them (Fig. 7a to c). We suggest that the CAB-like structures contain a global repressive signal (which may be associated with *pum1* transcripts). Since these structures are no longer present in newly formed PGCs (B7.g), a switch from transcriptional silencing in the germ line precursor cells to zygotic genome activation in newly formed PGCs may occur (Fig. 7c to d). Our observation of zygotic *vas4* transcription in the newly formed PGCs (B7.g cells) supports this model.

Our observation of large *pum1-*containing structures outside the embryos during late embryogenesis was unexpected. A corollary to our model is that somatic gene expression continues to be repressed in the somatic daughter cells (B7.s) as long as these structures are present (Fig. 7d). Hence, shedding the *pum1-*containing structures may be a mechanism allowing for initiation of zygotic transcription in the B7.s cells. For example, the PUF protein family member PUF-8 in *C. elegans* is thought to act as a repressor of the somatic transcription factor PAL-1 [24]. Maternally inherited *pal-1* transcripts, found in the germ line stem cells are not being translated when PUF-8 protein is present. One likely possibility is that the PUF protein, PUM1, acts similarly as a cell fate regulator by inhibiting translation of maternal transcripts encoding somatic transcription factors in the germ line progenitor cells.

In ascidians, the function of the somatic endodermal strand cells (B8.11 cells), which inherit the CAB remnants and contain numerous postplasmic/PEM transcripts, remains enigmatic [5]. In fact, the fate of CAB remnants in ascidians is not known. It would therefore be of interest to investigate if these remnants are shed from the B8.11 cells.

## Conclusions

Our data support the finding that the free-swimming tunicate *O. dioica* and the sessile ascidians employ a similar strategy for specifying germ line. We propose that the CAB and CAB-like structures have a key role in orchestrating the segregation of postplasmic components (including RNA and proteins) during germ-soma segregation in both ascidians and larvaceans.

## Methods

### Animal cultures and embryos

Animals were raised and maintained in laboratory culture at 15 °C. Mature animals were transferred to small dishes. Eggs and sperm were collected from mature animals that have completed spawning. In vitro fertilization was performed with diluted sperm solution. The embryos were developing at 18–20 °C and collected when they had reached the proper developmental stages.

### RNA probes and DNA constructs

Total RNA was isolated from oocytes using the RNeasy MiniKit (Promega) according to manufactures protocol. cDNA synthesis was performed with 0.5 μg total RNA and primed with random decamers using SuperScript® III reverse transcriptase (Invitrogen). PCR was performed with BioTaq DNA polymerase (Bioline) the amplified fragments were purified using the Qiaquick Gel extraction Kit (Qiagen) and cloned into the in vitro transcription vector pGEM® T-Easy (Promega). Linearized templates were prepared, and in vitro transcription reactions performed in the presence of digoxigenin DIG or fluorescein RNA Labeling Mix (Roche Applied Science). Accession numbers for genes involved in this study: *vas1* (CBY22958.1), *vas2* (CBY18436.1), *vas3* (CBY23797.1), *vas4* (CBY13641.1), *pum1* (CBY19123.1), and *pum2* (CBY18164.1).

### In situ hybridization

In situ hybridization was carried out according to protocol in [25] with the following modification. The hybridization buffer contained 50% formamide, 6 x SSC, 5 X Denhardt’s solution, 1 mg/ml yeast tRNA, 0.1% Tween. The probe concentration was 1 ng/ μl. Hybridization was performed at 60 °C for at least 16 h. After the washing step - with washing solution III (0.5× SSC, 0.1% Triton X-100) was completed - the samples were incubated in RNAse buffer (10 mM Tris-HCl pH 7.5, 0.5 M NaCl, 1 mM EDTA) for 5 min. Then the embryos were treated with RNase A (20 μg/ml in RNase buffer) for 30 min at 37 °C followed by two washing steps in RNase buffer for 30 min at 60 °C. The blocking buffer contained 1 x malic acid buffer pH 7.5, 1% blocking solution (Roche Applied Science), (0.1% Tween-20). Anti-DIG AP antibody (Roche Applied Science) was diluted 1:3000 in blocking buffer. Stained embryos were mounted in 50% glycerol/phosphate-buffered saline solution containing 0.1% Tween (PBT) or in Slow Fade ® Gold antifade reagent with DAPI (Invitrogen). Images were acquired using a Nikon Eclipse E800 microscope equipped with a Nikon DS-F1 digital camera and the software NIS-Elements F3.2.

### Immunohistochemistry

Embryos were incubated in fixative (4% paraformaldehyde/0.1 M MOPS/0.5 M NaCl) overnight at 4 °C. Following fixation, the embryos were washed 3 x in PBS and then treated by pronase (1:200 dilution of 10% pronase in PBS) for 2 min at RT. Pronase-treated animals were incubated in fixative for 20 min at RT. After the pronase treatment, the embryos were washed once in PBSTE (1× PBS/0.2% Tween/1 mM EDTA), three times in PBESTG (PBSTE/0.1% glycine), and twice in PBSTE. Blocking was performed in 3% BSA/PBSTE overnight at 4 °C. Then the embryos were incubated with an H3S28 antibody from Abcam (ab10543, at a 1:100 dilution in blocking buffer) for 6 days at 4 °C. After antibody incubation, the embryos were rinsed 6 x in PBSTE and the fixation step and the washing steps with PBSTE, PBSTEG, and PBSTE were repeated. The secondary antibody used were goat anti-rabbit IgG H&L (Alexa Fluor 568) antibody from Abcam (ab175471, at a 1:100) and the incubation step performed for 6 days at 4 °C. After this step, the embryos were washed 3 x in PBSTE. To visualize cell membranes, the embryos were incubated with Alexa Fluor 633 Phalloidin from Thermo Fisher Scientific for 1 h at RT. The samples were rinsed 3 x in PBT and mounted in Slow Fade ® Gold antifade reagent with DAPI (Invitrogen). Sequential imaging was performed using the Leica TCS SP5 confocal laser scanning microscopy and the software LAS-AF.

### Transmission electron microscopy

Electron microscopy studies were performed similar to [26]. Accordingly, sample preparation procedures were performed at room temperature. Sixteen-cell stage embryos were fixed in 2.5% glutaraldehyde/0.1 M sodium cacodylate/0.24 M NaCl (pH 7.4). Following rinsing in 0.1 M sodium cacodylate/0.24 M NaCl (pH 7.4), embryos were post-fixed in 1% OsO_4_/0.1 M sodium cacodylate/0.24 M NaCl (pH 7.4) and rinsed in distilled water. Subsequently, specimens were *en bloc* stained in 2% OsO_4_/1.5% potassium ferricyanide/0.1 M sodium cacodylate (pH 7.4). Following rinsing in distilled water embryos were incubated in 2% aqueous uranyl acetate and were gradually dehydrated in ethanol before exchanged to propylene oxide. For embedding the TAAB Araldite 502/812 kit was used, in which mixture A consisted of Embed-812/Araldite 502/DDSA (Dodecenyl Succinic Anhydride). To embed, propylene oxide was replaced by successive incubations of 1:3, 1:1, and 3:1 propylene oxide:mixture A, before overnight incubation in pure mixture A. Samples were finally embedded in fresh medium consisting of Embed-812/Araldite 502/ DDSA/ BDMA (Benzyl Dimethyl Amine) and polymerized at 60 °C for 48 h. Ultrathin sections (70 nm) were cut with a Leica EM UC7 ultramicrotome and counterstained with 2% uranyl acetate and lead citrate. Images were acquired on a Jeol JEM-1230 transmission electron microscope equipped with a *1 k × 1 k* CCD camera (Gatan multiscan).

## Additional files

Additional file 1: *pum1* is not a PGC marker in *O. dioica*. Color-enhanced version of Fig. 4d-f. In situ labelling with fluorescent-labeled RNA probe for *pum1* showing signal outside of the embryos prior to hatching. Panels D-F are the same as in the main Fig. 4. Below is shown a color-enhanced version of the same panels (D*-F*). Photoshop was used to extract the red colors which were then changed to magenta and superposed on the original images. The intensely *pum1*-staining object is indicated with triangles. (PDF 5201 kb)

Additional file 2: Detection of two large refractive structures in one of the B6.4 cell at gastrulation stage. In situ hybridization was performed with a DIG-labeled RNA probe. Developmental stage: An embryo at late gastrulation stage (A). Close-up views of the two B6.4 cells containing *pum1* transcripts (panels B and C). In panel B, the circumference of the two B6.4 cells are marked in white. In panel C, visible nuclei are marked by asterisks. White asterisks are used where two nuclei are observed within one B6.4 cell, while a black asterisk is used in the cell where only one nucleus can be observed. (PDF 1257 kb)

Additional file 3: Antibody staining of an epitope localized to the postplasm in the germ line precursor line. Confocal microscopy of embryos labelled for microfilaments (green), DNA (blue), and phosphorylated histone H3 (phosphorylated serine 28) (red). Only the overlays are shown here. The developmental stages are: A 4 cell-stage embryo (A). Animal view of an 8-cell stage embryo (B). A 16- cell stage embryo (C). In panel A and B, the antibody recognizes an epitope of the postplasm plus condensed phosphorylated chromosomes. The arrow head points to the non-chromosomal subcellular domain recognized by the H3S28 antibody. (PDF 2029 kb)

## Abbreviations

CAB: centrosome-attracting body
cER: cortical endoplasmatic reticulum
*O. dioica*: *Oikopleura dioica*
PEM transcripts: *posterior end mark* transcripts
PGCs: primordial germ cells
*pum*: *pumilio*
PVC: posterior vegetal cytoplasm/cortex
*vas*: *vasa*
