# Supplementary figures and images for: Evidence for a centrosome-attracting body like structure in germ-soma segregation during early development, in the urochordate Oikopleura dioica
Source: BMC Dev Biol. 2018 Feb 27;18:4. doi: 10.1186/s12861-018-0165-5 (PMC5830320; doi:10.1186/s12861-018-0165-5)

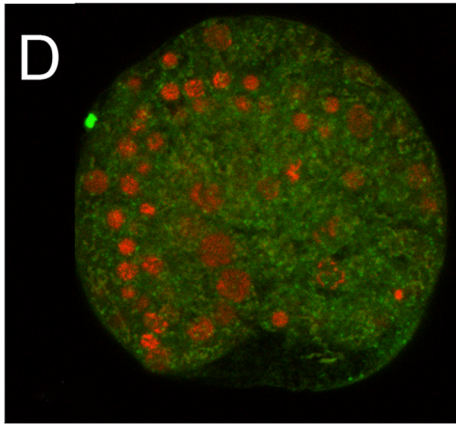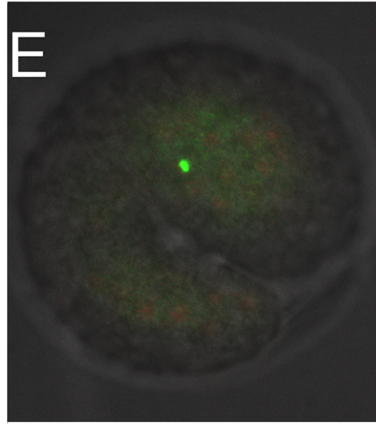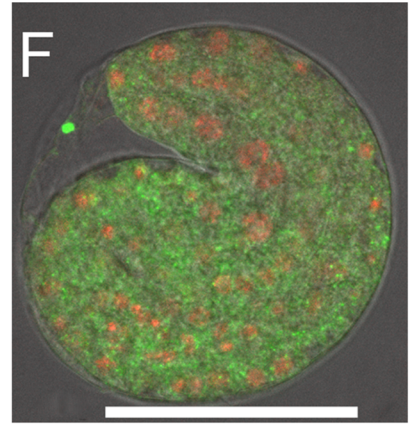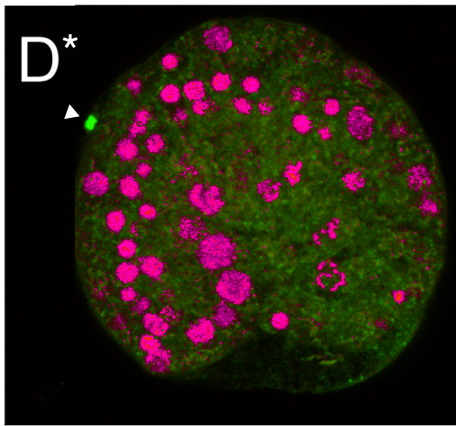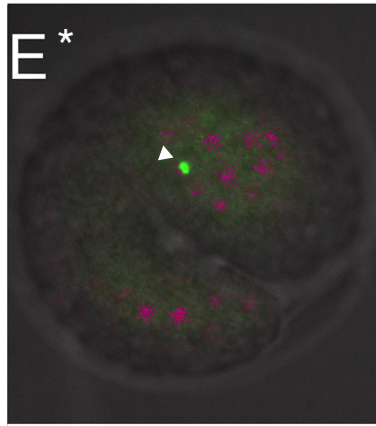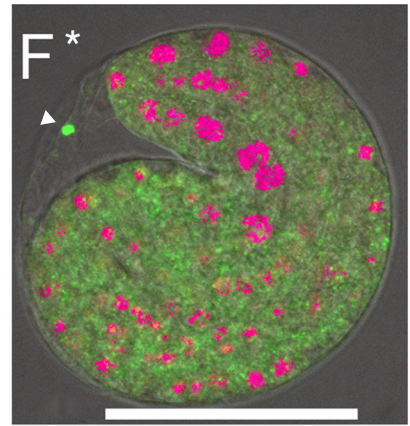

Supplement: Supplementary file 1 — pum1 is not a PGC marker in O. dioica. Color-enhanced version of Fig. 4d-f. In situ labelling with fluorescent-labeled RNA probe for pum1 showing signal outside of the embryos prior to hatching. Panels D-F are the same as in the main Fig. 4. Below is shown a color-enhanced version of the same panels (D*-F*). Photoshop was used to extract the red colors which were then changed to magenta and superposed on the original images. The intensely pum1-staining object is indicated with triangles. (PDF 5201 kb) [file 12861_2018_165_MOESM1_ESM.pdf]

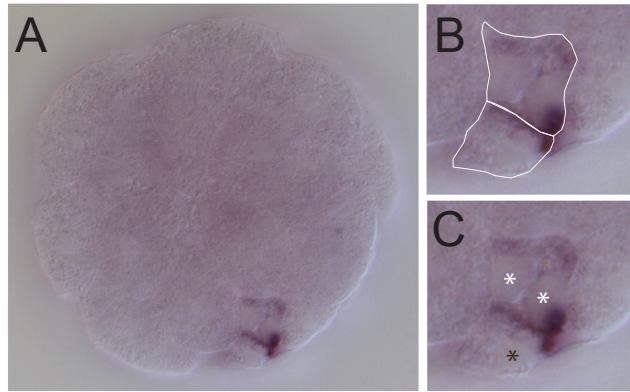

Supplement: Supplementary file 2 — Detection of two large refractive structures in one of the B6.4 cell at gastrulation stage. In situ hybridization was performed with a DIG-labeled RNA probe. Developmental stage: An embryo at late gastrulation stage (A). Close-up views of the two B6.4 cells containing pum1 transcripts (panels B and C). In panel B, the circumference of the two B6.4 cells are marked in white. In panel C, visible nuclei are marked by asterisks. White asterisks are used where two nuclei are observed within one B6.4 cell, while a black asterisk is used in the cell where only one nucleus can be observed. (PDF 1257 kb) [file 12861_2018_165_MOESM2_ESM.pdf]

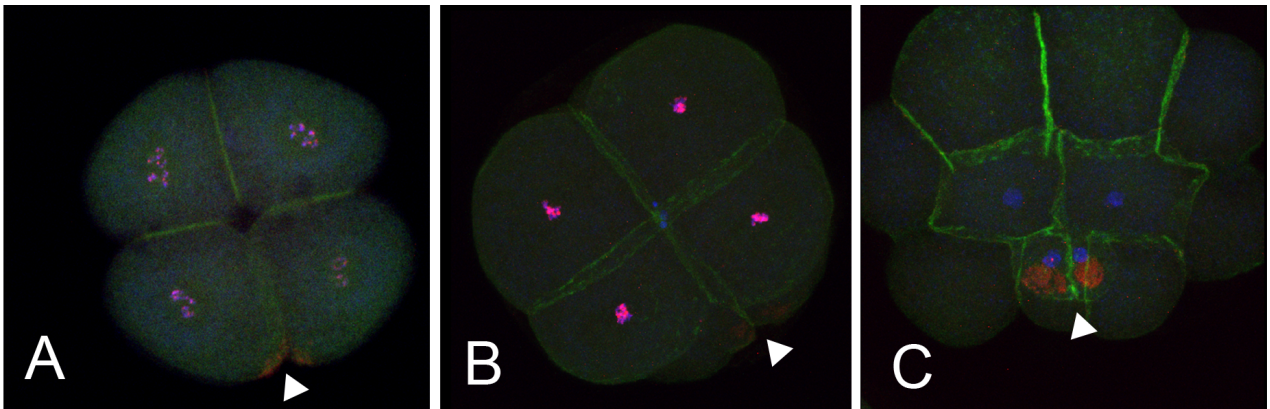

Supplement: Supplementary file 3 — Antibody staining of an epitope localized to the postplasm in the germ line precursor line. Confocal microscopy of embryos labelled for microfilaments (green), DNA (blue), and phosphorylated histone H3 (phosphorylated serine 28) (red). Only the overlays are shown here. The developmental stages are: A 4 cell-stage embryo (A). Animal view of an 8-cell stage embryo (B). A 16- cell stage embryo (C). In panel A and B, the antibody recognizes an epitope of the postplasm plus condensed phosphorylated chromosomes. The arrow head points to the non-chromosomal subcellular domain recognized by the H3S28 antibody. (PDF 2029 kb) [file 12861_2018_165_MOESM3_ESM.pdf]
